# Synthesis of Ag_3_PO_4_/Ag_4_P_2_O_7_ by microwave-hydrothermal method for enhanced UV–visible photocatalytic performance

**DOI:** 10.1038/s41598-022-26442-1

**Published:** 2023-03-23

**Authors:** Surin Promnopas, Wonchai Promnopas, Wachiraporn Maisang, Surangkana Wannapop, Titipun Thongtem, Somchai Thongtem, Orawan Wiranwetchayan

**Affiliations:** 1 Department of Physics and Materials Science, Faculty of Science, Chiang Mai University, Chiang Mai, 50200 Thailand; 2 Faculty of Science, Energy and Environment, King Mongkut’s University of Technology North Bangkok, Rayong Campus, Rayong, 21120 Thailand; 3 Materials Science Research Center, Faculty of Science, Chiang Mai University, Chiang Mai, 50200 Thailand; 4 Research Center in Physics and Astronomy, Faculty of Science, Chiang Mai University, Chiang Mai, 50200 Thailand

**Keywords:** Materials science, Nanoscience and technology

## Abstract

Ag_3_PO_4_/Ag_4_P_2_O_7_ photocatalysts were successfully synthesized by microwave-hydrothermal method. Tuning the properties of photocatalysts was achieved using different amount of acetic acid (CH_3_COOH) and sodium hydroxide (NaOH) to adjust pH value of precursor solution (pH = 4, 7, 10 and 12). The crystal structure, morphology and optical property of samples were characterized and explained. The photocatalytic activity of sample was determined by degradation of rhodamine B (RhB) and methyl orange (MO) under a wavelength range of 350–700 nm irradiation. The results demonstrated that the change in shape of particles was not observed whereas the average particle size was decreased with increasing pH value because of the high hydroxide ions (OH^−^). The sample synthesized in the solution with the pH of 10 exhibited excellent photocatalytic performance and stability because of the highest surface area and the present of Ag_4_P_2_O_7_ on the surface of particles. The highest photodegradation efficiency was 99.34 and 96.12% by degrading RhB and MO, respectively. The enhancement of photocatalytic performance of Ag_3_PO_4_/Ag_4_P_2_O_7_ was discussed. The active species trapping experiments showed that the h^+^ was the main active species to decompose the dye molecules.

## Introduction

Photocatalytic technology has attracted much attention for application on completely degrading organic contaminates in wastewater. Many novel semiconductor photocatalysts have been studied for photocatalytic utilization, such as TiO_2_^1^, BiOBr^2^, WO_3_^3^, LaFeO_3_^4^, BiPO_4_^5^, CeO_2_^6^, Ag_3_PO_4_^7^, ZnO^8^. Ag_3_PO_4_ is an one efficient photocatalyst for photocatalytic degradation because of its narrow band gap (2.39–2.54 eV)^9–12^ and positive valence potential^13^. Thus, the electron-hole pairs are easily generated under visible light leading to excellent photocatalytic oxidation ability. Nevertheless, the particle size of Ag_3_PO_4_ remains relatively large and low surface area which can hinder its performance in photocatalytic activity^14^.

The performance of photocatalyst depends on external and internal factors in the photocatalytic process: the external factors are intensity of light source, temperature, pH of dye solution and ratio of organic dyes to catalyst, and internal factors are crystal structure, optical properties, morphology, and magnetic behavior. Thus, external factors can be controlled during the experiment for better photocatalytic performance. Several researchers have focused on the method and adjusting certain factors for synthesizing Ag_3_PO_4_ photocatalyst to improve internal factors. Xu et al. synthesized Ag_3_PO_4_ by a simple precipitation method with different amount of ammonium molybdates to enhance photocatalytic activity. The results showed that the ratio of ammonium molybdates affected to morphologies of Ag_3_PO_4_, its shape changed from cubic to nano-flake. Moreover, the photocatalytic activity of BPA depended on light absorption and obviously accelerated carrier transfer, which are associated with the increasing (111) crystal number and catalytic active sites^15^. Amornpitoksuk et al. prepared Ag_3_PO_4_ by precipitation method using Na_3_PO_4_, Na_2_HPO_4_, and NaH_2_PO_4_ as a precipitating agent. They found that the purity, morphology, and particle size of Ag_3_PO_4_ powder were affected by type of phosphate salt. The pure Ag_3_PO_4_ prepared by Na_2_HPO_4_ with pH < 10 showed the highest photocatalytic efficiency to degrade methylene blue and rhodamine B, while the Ag_3_PO_4_ prepared by Na_3_PO_4_ showed the secondary phase as Ag_2_O with low active surface area led to decrease photocatalytic activity^16^. Futihah et al. synthesized Ag_3_PO_4_ by coprecipitation method under different pH value. The morphology of Ag_3_PO_4_ was changed due to the change of the surface energy. The Ag_3_PO_4_ prepared at pH 11 showed higher amount of the tetrahedron structure with the smaller particle size leading to highest activity for degraded RhB^17^. Therefore, morphology, crystalline structure, and particle size of Ag_3_PO_4_ catalyst are the important key to enhance the photodegradation activity.

Microwave hydrothermal method is a hybrid process of microwave and hydrothermal combination. Microwaves radiation promoted a fast and homogeneous heating of system leading to high reaction rate and acceleration of crystal growth^18,19^. Thus, the method has been considered as strategy for preparation of semiconductor nanoparticles with uniform morphology. In this study, the Ag_3_PO_4_/Ag_4_P_2_O_7_ photocatalysts powders were prepared by microwave hydrothermal method. Acetic acid and NaOH were added into precursor solution to adjust the pH value. The effect of pH value on morphology, crystal structure and optical property was investigated and explained. The photocatalytic activities of photocatalysts were studied through degradation of RhB and MO under the wavelength range of 350–700 nm irradiation. The active species trapping, stability, and recyclability of photocatalysts were also studied and discussed.

## Methods

### Preparation of photocatalyst

Photocatalyst was synthesized by microwave hydrothermal method, 6 mmol of Silver Nitrate (AgNO_3_) and 2 mmol of Di-Sodium Hydrogen Phosphate Dehydrate (Na_2_HPO_4_ • 2H_2_O) were dissolved in 35 ml deionized water (DI) under continuous stirring for 180 min at room temperature. Then, acetic acid (CH_3_COOH) was added to the mixture till the solution reached pH 4. The same procedures were repeated to prepared precursor solutions having different pH 7, 10 and 12 by adding sodium hydroxide (NaOH). After that these solutions were transferred to glass autoclaves with irradiated by 270 W microwave for 30 min. After cooling down to room temperature, the as-synthesized products were filtered by filter paper and washed with DI water and ethanol for several times. Finally, the final products were dried at 80 °C for 12 h. The samples prepared with pH 4, 7, 10 and 12 were marked as Ag -pH4, Ag -pH7, Ag -pH10 and Ag -pH12, respectively.

### Characterization

Morphology of the samples was characterized by field emission scanning electron microscopy (FE-SEM, JEOL JSM-6335F) operating at 15 kV. The specific surface area was evaluated by the Brunauer–Emmett–Teller (BET) nitrogen adsorption isotherm (Quantachrome Model Autosorb 1MP). The phase and crystalline structure were characterized by X-ray diffraction (XRD; Rigaku MiniFlex600) with Cu-Kα radiation (λ = 1.5418 Å). The species and chemical state were characterized by X-ray photoelectron spectroscopy (XPS; AXIS Ultra DLD, Krator Analytical Ltd.) which was calibrated by C 1 s level from carbon samples at 284.5 eV. The optical property of samples was studied by a UV–Visible diffuse reflectance spectroscopy (UV-DRS; Shimudzu UV 2006) in the wavelength range of 200–800 nm. Raman spectra was characterized by Raman spectroscopy (T64000 HORIBA Jobin Yvon) using a 50 mW and 514.5 nm wavelength Ar green laser. Fourier transform infrared spectroscopy (FT-IR; Bruker Tensor 27) operated in the range of 400–2000 cm^−1^.

### Photocatalytic activitiy

Photocatalytic activities of the samples were evaluated by the photocatalytic decomposition of rhodamine B (RhB) and methyl orange (MO) under a wavelength range of 350–700 nm irradiation (35 W Xe lamp). The average light intensity for photocatalytic testing was 74,500 Lux. The 0.25 g of photocatalyst was susped in 250 ml dye solutions under continuous stirring and kept in the dark for 30 min to establish an adsorption–desorption equilibrium. The 3 ml of dye suspension was collected every 5 min. After the first run, the photocatalysts were separated by centrifuged from the suspension and then washed with deionized water. After drying at 80 °C, the recovered catalysts were reused for the next run under the same experimental conditions. The concentration of dye suspension was then investigated by a UV–Vis absorption spectrometer at λ_max_ = 464 and 554 nm for MO and RhB, respectively. The degradation efficiency (%) was calculated by the follwing equation:^1,7^.

1 $${\text{Degradation}}\;{\text{efficiency}}\;\left( \% \right) = \left( {\frac{{{\text{C}}_{0} - {\text{C}}_{t} }}{{{\text{C}}_{0} }}} \right) \times 100\%$$

where C_0_ is the initial concentration of dye and C_t_ is the concentration of dye when the photocatalysis was proceeding within the elapsed time (t). Additionally, Isopropyl alcohol; IPA (OH^•^), p- Benzoquinone; BQ (^•^O_2_^−^) and EDTA (h^+^, holes) were used as scavenger to explain the photocalytic mechanism. The stability and recyclability of Ag_3_PO_4_ catalysts were also studied.

## Results and discussion

**Figure 1:**
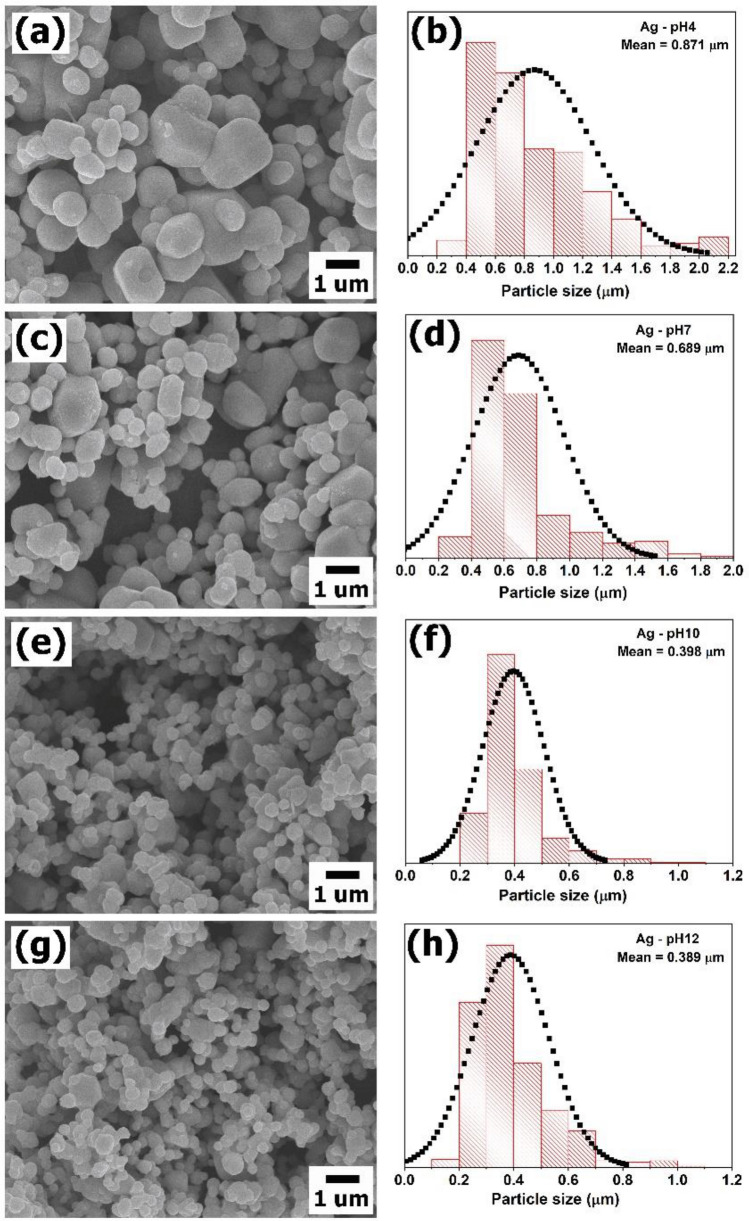
SEM image and average particle sizes by histogram of (**a**, **b**) Ag-pH4, (**c**, **d**) Ag-pH7, (**e**, **f**) Ag-pH10 and (**g**, **h**) Ag-pH12, respectively.

The morphology of samples synthesized by microwave hydrothermal method at different pH values was studied by FE-SEM. For all samples, the particle showed irregular shape with different the particle size, as shown in Fig. 1. Hydroxide ions (OH^−^) are a morphology controlling agent which affects nucleation and growth of particle. The Ag -pH4 prepared by adding acetic acid (pH = 4) in solution showed the largest average particles size of 871.44 nm. With addition of NaOH in precursor solution (pH = 7, 10, 12), OH^−^ ions could absorb on the surface of primary particles leading to an increase electrostatic repulsion among primary particles^20^. Therefore, the average particle size reduced because of higher OH^−^ ions concentration. Moreover, the solution reached supersaturation state by adding NaOH when supersaturation of Ag_3_PO_4_ nuclei occurred, resulting the nuclei form rapidly and therefore smaller particles^21,22^. The average particle size and the specific surface area of samples are summarized in Table 1. Ag-pH10 presented highest the specific surface area of 7.77 m^2^/g which it is very important for the photocatalytic activity.

**Table 1:** Average crystallite, particle size, surface area, the degradation efficiency and band gap energy (${E}_{g}$) of photocatalysts.

Sample	Crystallite size (nm)	Particle size (nm)	Surface area (m^2^/g)	Degradation efficiency of RhB (%)	Degradation efficiency of MO (%)	Ag_4_P_2_O_7_		
						${E}_{g}$(eV)	${E}_{CB}$ (eV/NHE)	${E}_{VB}$ (eV/NHE)
Ag-pH 4	136.30	871.44	6.41	99.08	94.81	–	–	–
Ag-pH 7	129.23	689.72	6.97	98.70	95.70	2.72	+ 0.26	+ 2.98
Ag-pH 10	127.58	397.70	7.77	99.34	96.12	2.96	+ 0.14	+ 3.10
Ag-pH 12	104.13	388.58	7.57	99.26	93.65	3.08	+ 0.08	+ 3.16

Conduction band edge potential (${E}_{CB}$) and valence band edge potential (${E}_{VB}$) of Ag_4_P_2_O_7_.

**Figure 2:**
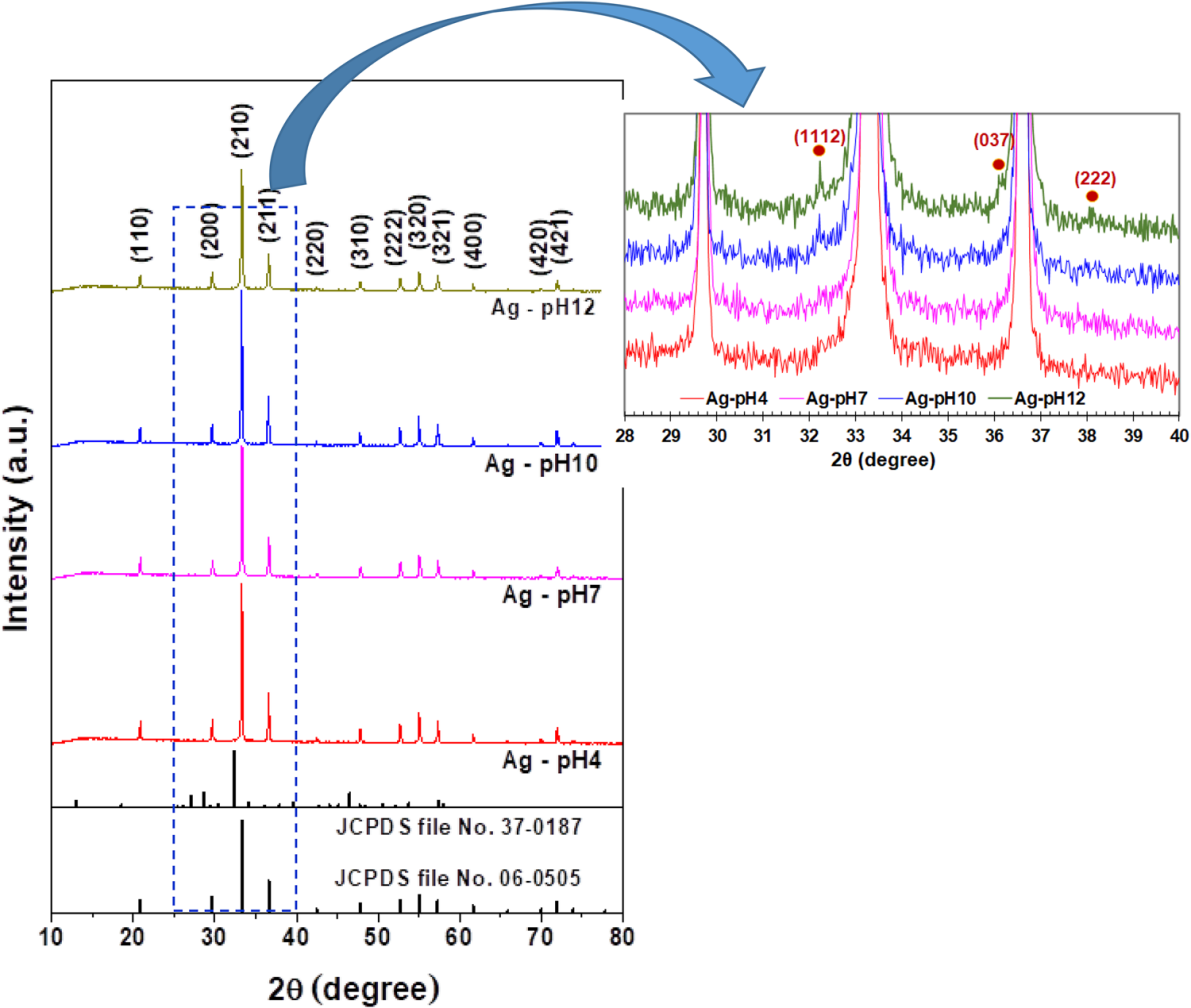
XRD patterns of samples with different pH values.

Figure 2 shows the XRD patterns of samples with different pH values. The diffraction peaks of samples at 2θ = 20.1, 29.7, 33.3, 42.5, 47.8, 52.7, 55.0, 57.3, 61.6, 70.0, and 71.9° corresponded to the body-centered cubic Ag_3_PO_4_ (JCPDS database No. 06-0505)^7,23^. However, small peaks of Ag_4_P_2_O_7_ were found for the synthesis starting at a high of pH solution. The very weak peaks at 2θ = 32.3, 36.1 and 38.1° corresponded to the hexagonal Ag_4_P_2_O_7_ (JCPDS database No. 37-0187). The average crystallite sizes can be calculated from XRD analysis at the (210) plan by using the Debye Scherrer’s formula^24^ as follows:

2 $$D = \frac{K}{\beta \cos \theta }$$

where λ is the Cu Kα wavelength (1.5406 Å), K is the shape factor (0.9), *β* is the full width at half maximum (FWHM) and *θ* is the Bragg’s angle. The results have been summarized in Table 1.

**Figure 3:**
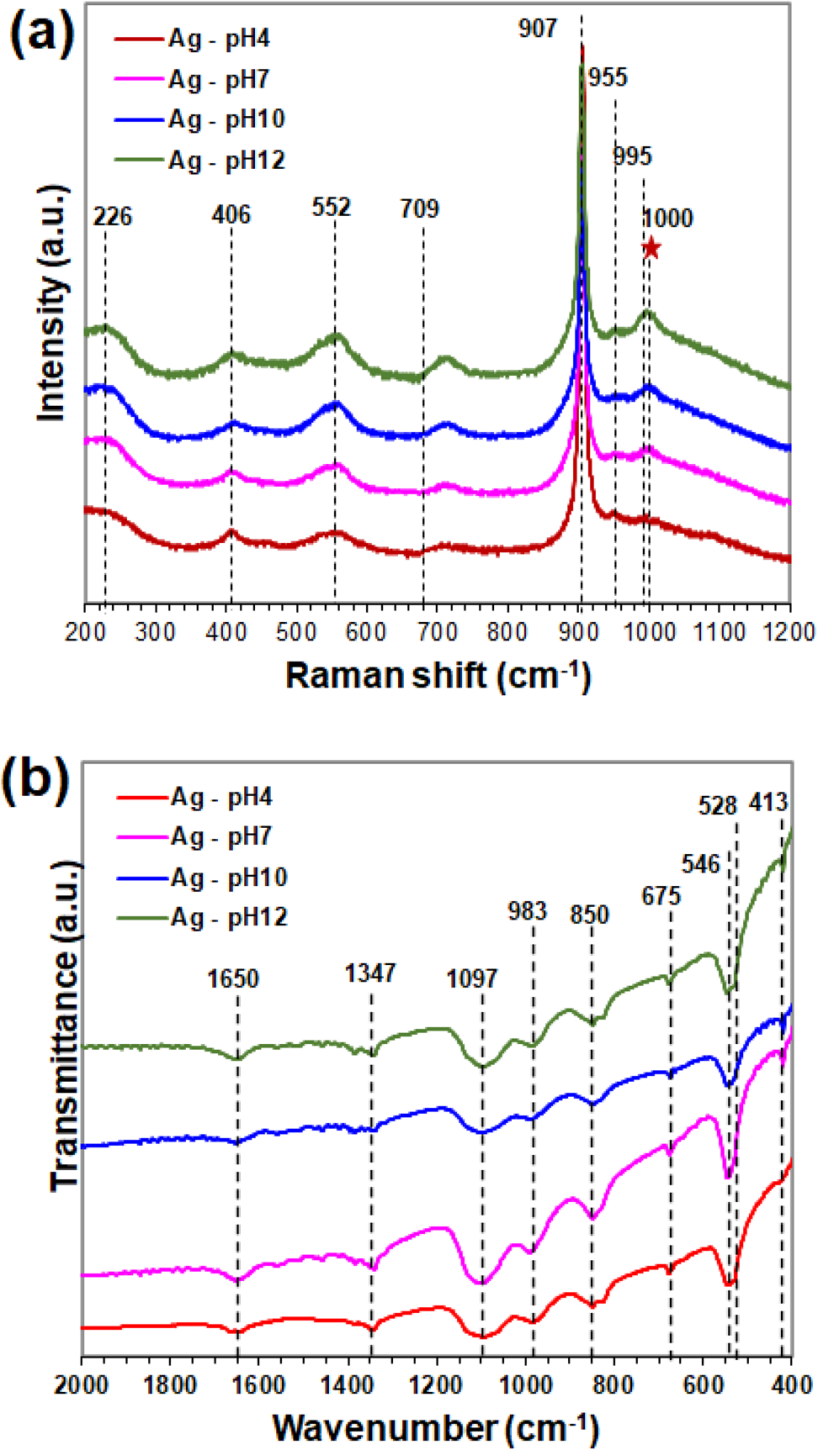
Raman spectra (**a**) and FT-IR spectra (**b**) of samples with different pH values.

Crystal structure of the samples was characterized by Raman spectroscopy over the range of 0–1200 cm^–1^, as shown in Fig. 3a. The Raman spectra of samples corresponded to the crystalline structure of cubic Ag_3_PO_4_ with P4-3n (218) space group were identified^25^. The strongest intensity peak at 907 cm^−1^ was attributed to the symmetrical terminal oxygen stretching modes of the PO_4_ cluster (A_1_)^26^. The weak peaks at 226 cm^−1^ assigned to rotation and/or translation vibration modes of PO_4_ cluster [T_2_]^25,26^, respectively. The two board peaks related to symmetric (E) and asymmetric (T_2_) bending modes of PO_4_ cluster were found at 406 and 552 cm^−1^, respectively. The very weak peaks at 709 and 955 cm^−1^ were corresponded to the symmetric stretching of O–P–O bonds (A_1_) or, alternatively, to combination mode and asymmetric stretching of O–P–O bonds (T_2_), respectively. The band at 995 cm^−1^ assigned to asymmetric stretching vibration of PO_4_ cluster (T_2_)^26^. The main peak of Ag_4_P_2_O_7_ was found at 1000 cm^−1^ assigned to symmetric vibration modes of PO_3_^27^, which the intensity of these peaks increased with increasing of pH. It means that the crystallinity or amount of Ag_4_P_2_O_7_ was increased. Other modes of Ag_4_P_2_O_7_ were not observed in this research may be because of their low intensities and/or overlapping bands. The contribution of Raman modes of Ag_4_P_2_O_7_ and Ag_3_PO_4_ were obviously noted in the composites. These results agreed with the XRD result analysis.

Furthermore, FTIR spectra of samples were performed at the range of 400–2000 cm^−1^ (Fig. 3b). The all of samples showed the peaks corresponded to the existence of Ag_3_PO_4_. The weak peak observed at 413 cm^−1^ was assigned to HPO_4_ vibration mode^28^. The strong peak at 528 and 546 cm^−1^ was ascribed to the O=P–O bending vibration modes^9,29^. For the band peak at 675 cm^−1^ in ranging 600–700 cm^−1^ could be attributed to the stretching vibration of O–P–O^30^. The absorption band at 850 and 1097 cm^−1^ was related to the symmetric and asymmetric stretching vibration of P–O–P ring, respectively^31^, while the peak at 983 cm^−1^ was related to P–O stretching vibration of phosphate^9^. The two peaks at 1347 and 1650 cm^−1^ were assigned to stretching vibration of P=O^32^ and H–O–H bending mode of water^7,31^, respectively.

**Figure 4:**
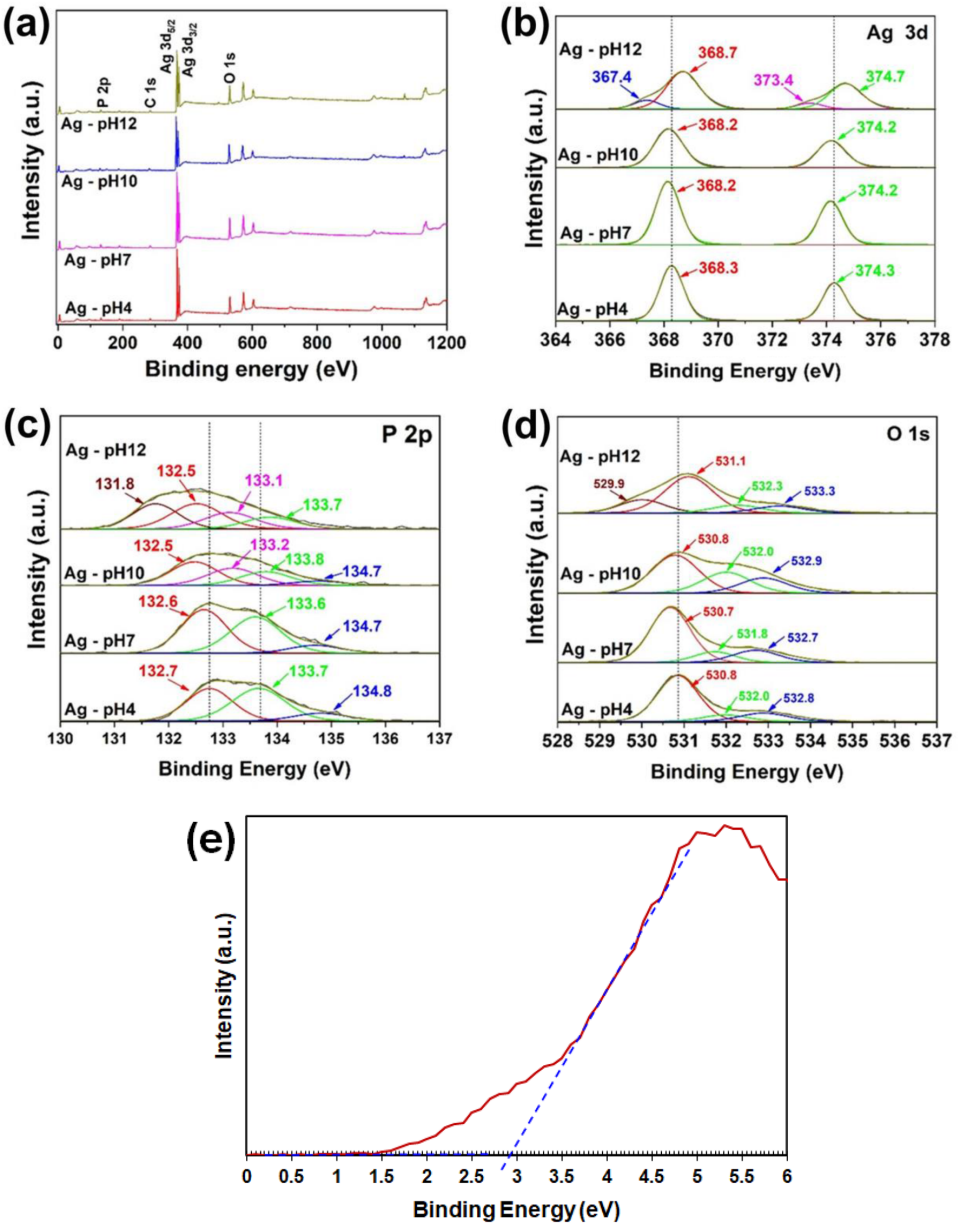
XPS spectra of samples for (**a**) survey scan, (**b**) Ag 3d, (**c**) P 2p and (**d**) O 1 s with different pH values. (**e**) The valence band XPS spectrum of Ag-pH4.

The oxidation states of all samples were carried out by the XPS measurement to confirm the surface composition and chemical state of samples synthesized with different pH value in precursor solution. The XPS survey spectrum (Fig. 4a) confirmed that samples consist of Ag, P and O elements. Figure 4b showed high-resolution XPS spectra of Ag 3d region. The peaks at binding energy (BE) in the range of 368.2–368.7 eV and 374.2–374.7 eV were attributed to Ag 3d_5/2_ and Ag 3d_3/2_ respectively, where the peaks are separated by 6.0 eV^33^. These results showed that the formal chemical valence state of Ag^+^ of Ag_3_PO_4_^33–35^. Additionally, the Ag-pH12 sample showed the peaks at 367.4 and 373.4 eV which related to the Ag^+^ state of Ag_4_P_2_O_7_^27^, while the samples prepared at low pH were not observed may be due to a low amount of Ag_4_P_2_O_7_ and/or the overlapping bands, as same as the results from XRD and Raman spectra. However, the peaks of Ag 3d_5/2_ and Ag 3d_3/2_ slightly shifted to lower BE may be because of the formation of Ag_4_P_2_O_7_. Figure 4c shows the XPS spectra of P 2p of as-preparing Phosphorus has five valence electrons. Therefore, the valence (x) can change from P^3+^ to P^x+^ (x < 5) with different the BE from 131 to 136 eV^36^. The peaks at 132.5–132.7 eV and 133.6–133.8 eV were associated with P2p_3/2_ and P2p_1/2_ respectively, indicating the formal chemical valence state of P was + 5 (P^5+^) containing in PO_4_^3‒^ of Ag_3_PO_4_^13,33,35,36^. Those peaks at 134.7–134.8 eV came from P^3+^ of H_3_PO_4_^36^. For pH = 10 and 12, the other peaks locating at 133.1–133.2 eV were the P–O–P bond (P^3+^) of Ag_4_P_2_O_7_^36,37^. The O 1 s spectrum of samples were shown in Fig. 4d. The peaks in the range of 530.7–531.1 were corresponded with oxygen lattices in the structure of Ag_3_PO_4._ The peaks in the range of 531.8–532.3 were assigned to O^2−^ anion on the surface of samples with the formation of defected or oxygen vacancies. The last peaks located at 532.7–533.3 eV were caused by the adsorbed –OH group of H_3_PO_4_ or water molecules on the surface^10,33,38^. He et al.^37^ studied the XPS spectra of Ag_3_PO_4_, Ag_4_P_2_O_7_, and Ag_3_PO_4_/Ag_4_P_2_O_7,_ they found that O 1 s peaks of Ag_3_PO_4_ and Ag_4_P_2_O_7_ were showed at 530.48 and 530.88 eV, respectively. Thus, the peaks related to oxygen state of Ag_4_P_2_O_7_ were not observed probably due to overlapping band. Additionally, the P 2p component at 131.8 eV and the O 1 s component at 529.9 eV that was found from Ag-pH 12 sample could be associated to the surface oxide or with oxidation state less than + 5 (P^x+^, x < 5)^22^. As the results from XPS spectra, the BE of Ag 3d and P 2p slightly shifted caused by the interaction of Ag_3_PO_4_ with Ag_4_P_2_O_7_. The Ag_4_P_2_O_7_ was obtained by adding NaOH into solution, which the concentration of OH^−^ increased, the reaction was more preferred to form Ag_4_P_2_O_7_. The synthesis reactions of Ag_3_PO_4_, Ag_4_P_2_O_7_ and H_3_PO_4_ are shown by the following formula.

3 $${\text{NaOH }} \rightleftharpoons {\text{ Na}}^{ + } + {\text{OH}}^{ - }$$

4 $${\text{AgNO}}_{3} { } \rightleftharpoons {\text{ Ag}}^{ + } + {\text{NO}}_{3}^{2 - }$$

5 $${\text{Na}}_{2} {\text{HPO}}_{4} { } \rightleftharpoons { }2{\text{Na}}^{ + } + {\text{HPO}}_{4}^{2 - }$$

6 $${\text{HPO}}_{4}^{2 - } + {\text{Ag}}^{ + } + {\text{OH}}^{ - } { } \rightleftharpoons {\text{Ag}}_{3} {\text{PO}}_{4} + {\text{H}}_{2} {\text{O}}$$

7 $$2{\text{HPO}}_{4}^{2 - } + 4{\text{Ag}}^{ + } + 2{\text{OH}}^{ - } { } \rightleftharpoons {\text{Ag}}_{4} {\text{P}}_{2} {\text{O}}_{7} + {\text{H}}_{2} {\text{O}} + 2{\text{H}}^{ + }$$

8 $${\text{HPO}}_{4}^{2 - } + 2{\text{H}}^{ + } { } \rightleftharpoons {\text{H}}_{3} {\text{PO}}_{4}$$

Figure 4e shows the valence band XPS spectrum of Ag-pH4. The ${E}_{VB}$ value was determined to be ~  + 2.92 eV.

**Figure 5:**
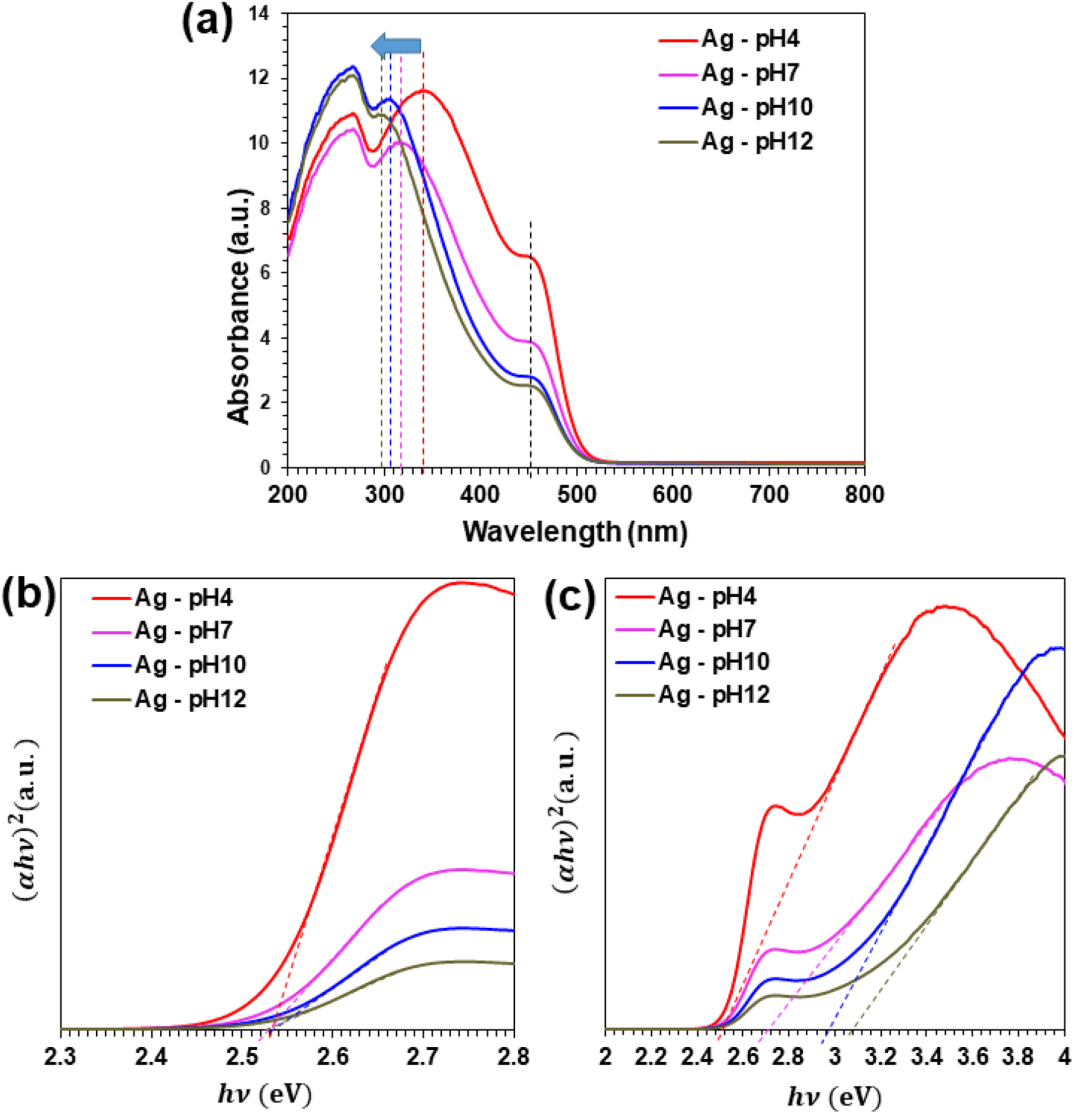
(**a**) The UV–vis absorption and (**b**, **c**) the plot of (αhν)^2^ versus hν of samples with different pH values.

The UV–vis absorption spectra of photocatalysts prepared with different pH values in precursor solution show in Fig. 5a. For all the samples, ${E}_{g}$ of Ag_3_PO_4_ was estimated to 2.53 eV (Fig. 5b). As the results, the visible light absorption intensities reduced in the 350–500 nm region and the absorption peak located at 340 nm (pH 4) shifted to 320 nm (pH 7), 305 nm (pH 10), and 298 nm (pH 12) as the increase of pH. These results had been caused by the coexistence of Ag_3_PO_4_ and Ag_4_P_2_O_7_. The direct band energy gap (${E}_{g}$) of Ag_3_PO_4_/Ag_4_P_2_O_7_ samples were estimated to 2.72, 2.96, and 3.04 eV in Fig. 5c. Normally, the ${E}_{g}$ of Ag_4_P_2_O_7_ is 3.36–3.42 eV^27,39,40^, suggesting that the Ag_3_PO_4_ reduced the excitation energy of Ag_4_P_2_O_7_. Therefore, the excessive Ag_4_P_2_O_7_ can interrupt the creation of photo-induced electrons and holes.

**Figure 6:**
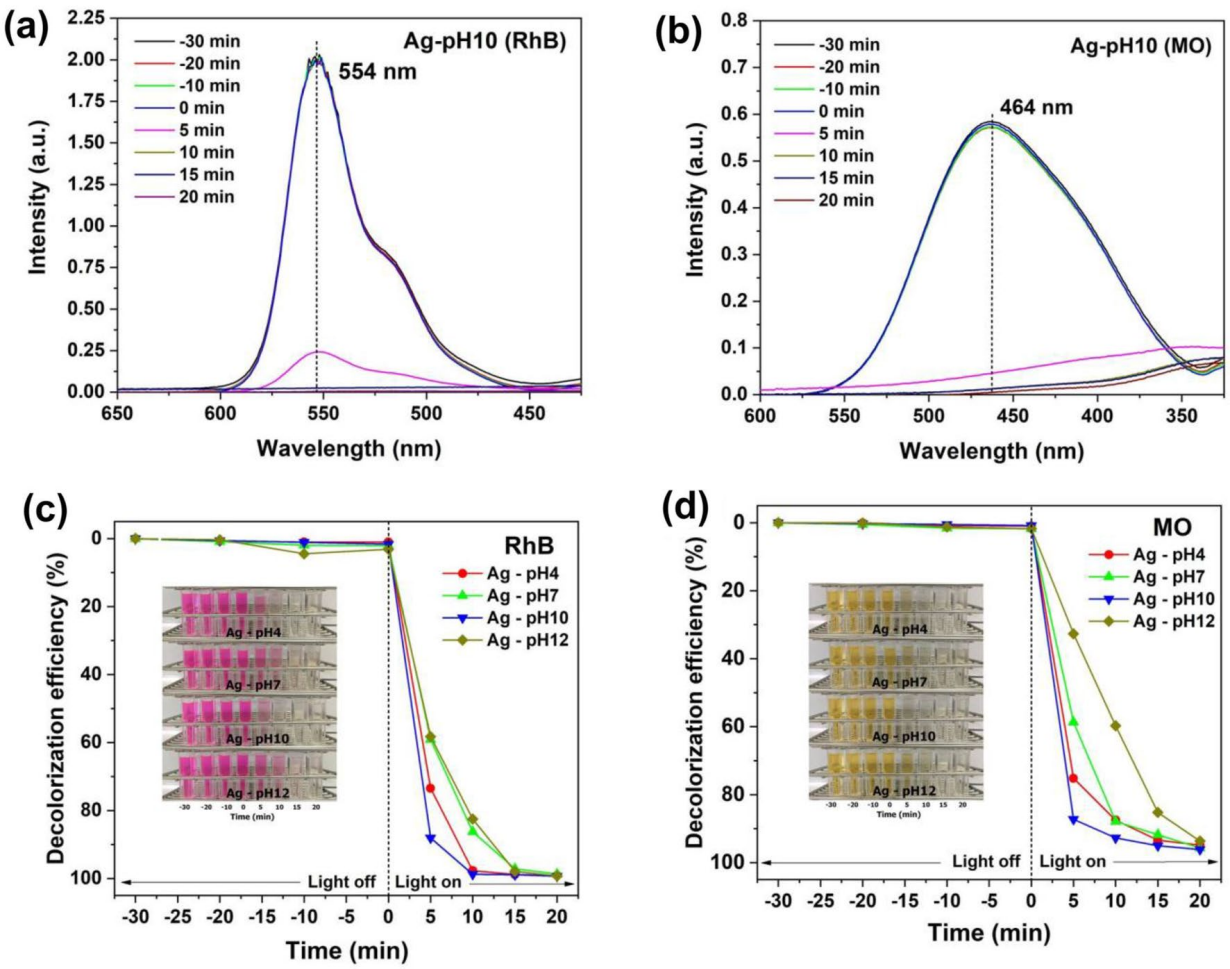
UV–vis absorption of (**a**) RhB and (**b**) MO in present of Ag-pH10 powder during irradiation time for 20 min. Photocatalytic degradation efficiency of (**c**) RhB and (**d**) MO of samples with different pH values.

The photocatalytic activity of samples was studied using the degradation of 2.0 × 10^−5^ M cationic RhB and anionic MO under a wavelength range of 350–700 nm irradiation. Before photodegradation, RhB and MO adsorption were measured in the dark for 30 min. The results showed that the concentration of RhB and MO was not changed, implying that the self-photolysis of MO and RhB was negligible^41^. The absorbance of RhB and MO in the present of Ag-pH10 powder rapidly decreased during irradiation within 20 min (Fig. 6a–b) leading to the changing in color of dye solutions from original color to colorless, indicated that the structure of RhB and MO was destroyed. Figure 6c–d show the photodegradation efficiencies of RhB and MO, the results are summarized in Table 1. Ag-pH10 exhibited highest photodegradation efficiencies of 99.34% for RhB and 96.12% for MO. The photodegradation performance are in sequence as follows: Ag-pH10 > Ag-pH4 > Ag-pH7 > Ag-pH12.

**Figure 7:**
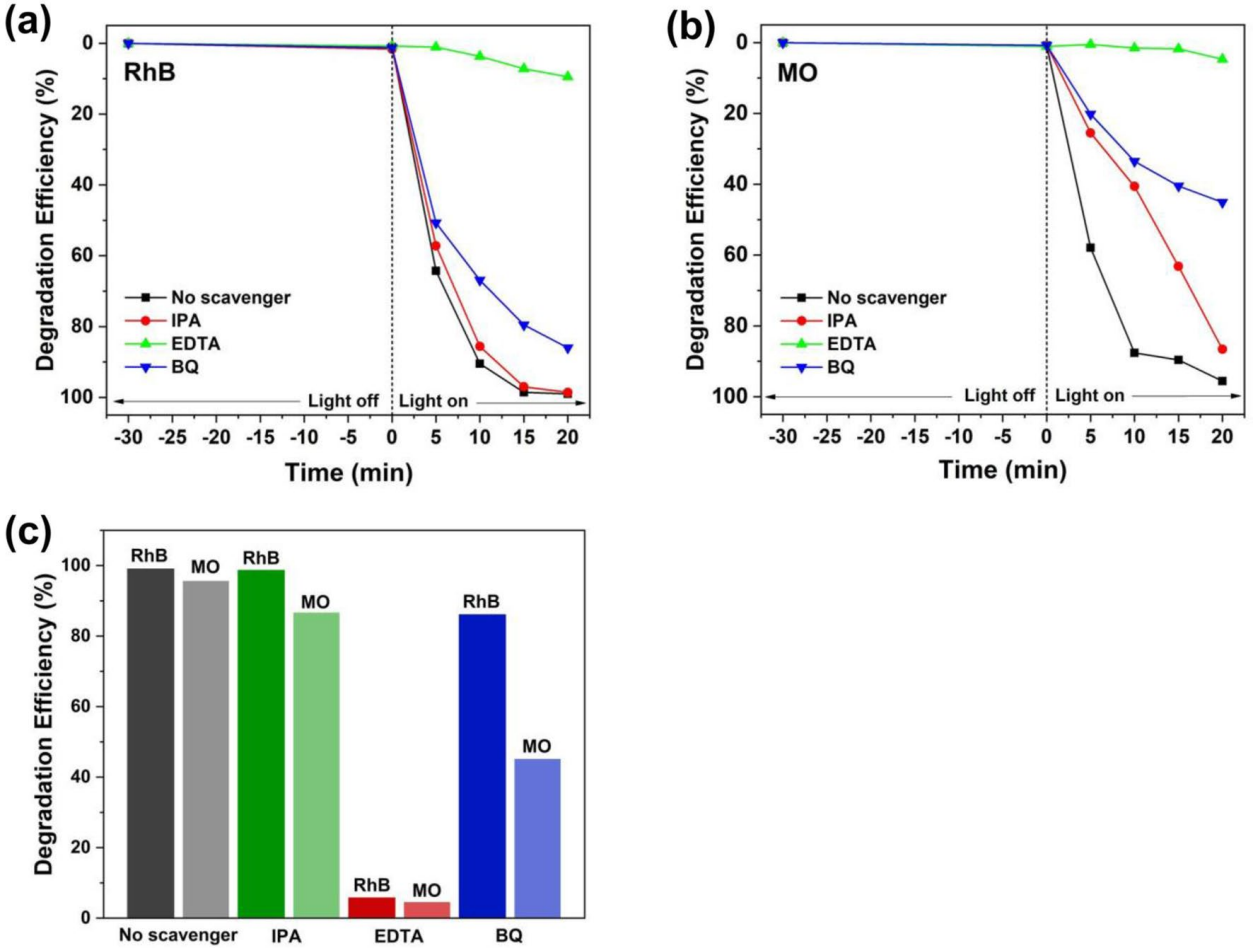
Decolorization efficiencies in presence of radical scavengers: IPA, EDTA and BQ for the degradation of (**a**) RhB and (**b**) MO by using Ag-pH10, and (**c**) comparison effect of different scavengers at 20 min irradiation.

Active species in the photocatalytic reaction of RhB and MO were then studied by isopropanol (IPA), p-benzoquinone (BQ) and ethylenediaminetetraacetic acid (EDTA) as hydroxyl radicals (•OH), superoxide redicals (•O_2_^−^) and holes (h^+^) scavengers, respectively. These scavengers and Ag-pH10 photocatalyst were added into the dye solution during 20 min irradiation time. The experiment results show in Fig. 7. The decolorization efficiencies were in sequence as follows: IPA > BQ > EDTA. The EDTA strongly interfered photocatalytic process mediated by Ag_3_PO_4_. Consequently, the h^+^ was the main active species as oxidative agents during the photocatalysis. Although the holes in the CB of Ag_3_PO_4_ can react with H_2_O to produce •OH redicals, •OH radicals were not the active species in the photocatalytic degradation process. Additionally, •O_2_^−^ was obviously involved in the degradation of dyes but the reaction rate was slow.

To explain the photocatalytic mechanism, the potential band structures of Ag_3_PO_4_ and Ag_4_P_2_O_7_ versus Normal Hydrogen Electrode (NHE) can be calculated by the Eqs. (9) and (10)^10^:

9 $$E_{VB} = \chi - E_{C} + 0.5E_{g}$$

10 $$E_{CB} = E_{VB} - E_{g}$$

where ${E}_{VB}$ and ${E}_{CB}$ are valence band edge potential and conduction band edge potential, respectively. is electronegativity of the semiconductor ($\chi$= 6.16 eV for Ag_3_PO_4_^10^ and $\chi$ = 6.12 eV for Ag_4_P_2_O_7_^27^). ${E}_{C}$ is energy of free electron on the hydrogen scale which is ~ 4.5 eV. The ${E}_{VB}$ and ${E}_{CB}$ value of Ag_3_PO_4_ were calculated to be + 2.92 (~ + 2.92 eV from the valence band XPS spectrum) and + 0.40 eV/NHE, respectively. For Ag_4_P_2_O_7_, the values are summarized in Table 1.

**Figure 8:**
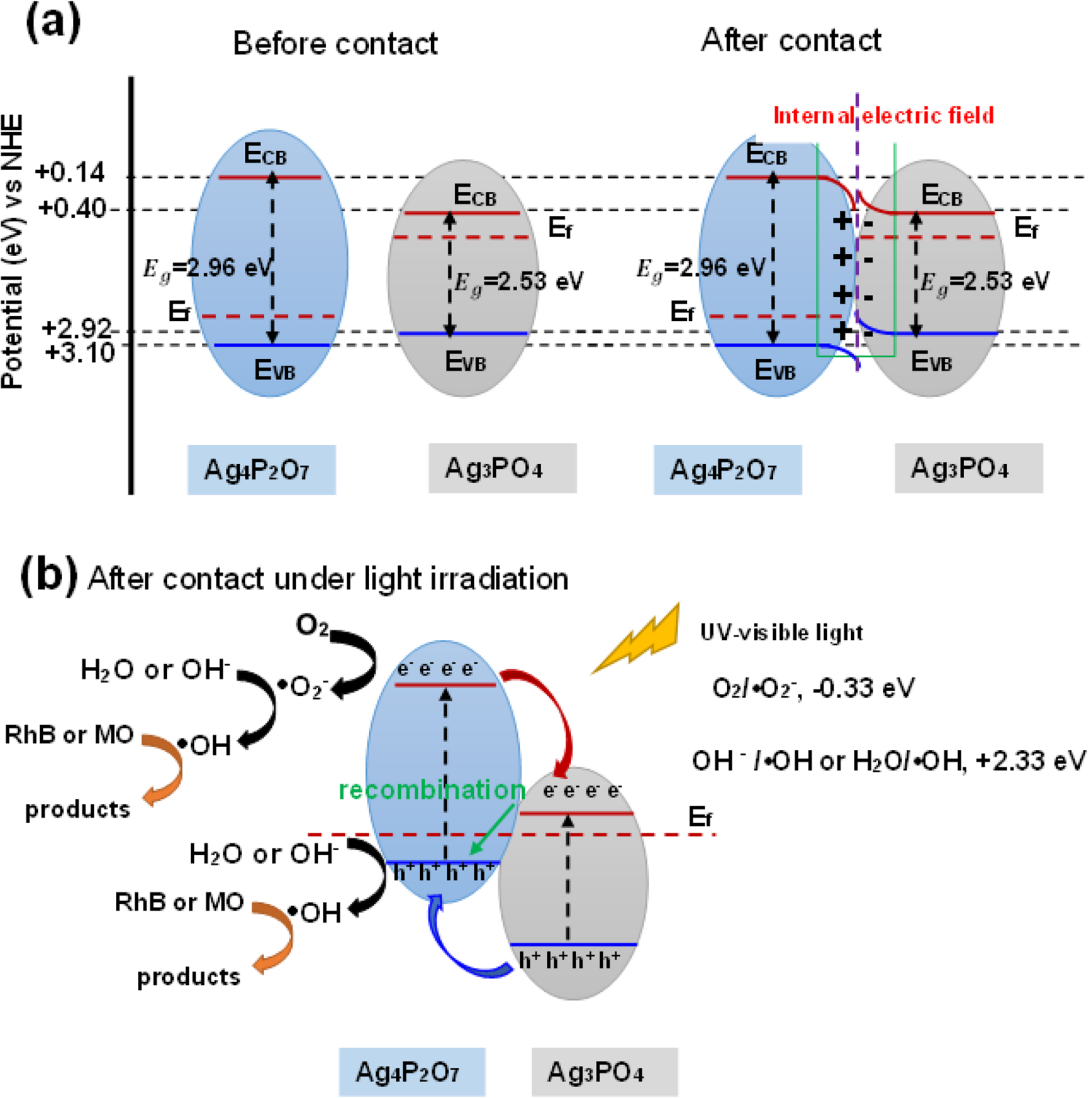
(**a**) S-Scheme heterojunction of Ag_3_PO_4_ and Ag_4_P_2_O_7_ before and after contact. (**b**) Schematic photocatalytic mechanism by using Ag-pH10 for degradation of dye under UV–visible irradiation.

Figure 8a shows S-Scheme heterojunction of the n-type Ag_3_PO_4_ and p-type Ag_4_P_2_O_7_ before and after contact. The valence band (VB) edge and Fermi level of isolated Ag_4_P_2_O_7_ were lower than that of Ag_3_PO_4_, while the conduction band (CB) edge was higher. After contacting, the interfacial charge transfer occurred. Electrons from Ag_4_P_2_O_7_ transferred to Ag_3_PO_4_ leading to Ag_4_P_2_O_7_ being negatively charged and Ag_3_PO_4_ being positively charged, the internal electric field at the interface region between these semiconductors was formed. The electrons could transfer until their Fermi level (E_f_) reached equilibrium. The band bending, production of the space-charge region, formation of depletion and accumulation layer occurred^42–44^. Thus, the band edge of Ag_4_P_2_O_7_ bended downward while the band edge of Ag_3_PO_4_ bended upward. Figure 8b shows the photocatalytic mechanism for the degradation of RhB and MO under UV–vis irradiation by using Ag -pH10. The electron–hole pairs were generated. The redox potential of O_2_/•O_2_^–^ (− 0.33 eV/NHE) was less negative than that of the CB of Ag_4_P_2_O_7_, which indicates that electrons could react with O_2_ to produce •O_2_^−^. Simultaneously, a part of electrons in the CB of Ag_4_P_2_O_7_ could transfer to the CB of Ag_3_PO_4_. Concomitantly, the h^+^ in the VB of Ag_3_PO_4_ also transferred to the VB of Ag_4_P_2_O_7_, which could enhance charge separation. The VB of Ag_3_PO_4_ (+ 0.39 eV/NHE) was more positive than that of the redox potential of H_2_O/•OH or OH^−^/•OH (+ 2.38 eV/NHE)^40^, the h^+^ could react with H_2_O or OH^−^ to produce •OH. However, the electrons in the CB of Ag_3_PO_4_ could recombine with the h^+^ in the VB of Ag_4_P_2_O_7_ lead to a decrease of photodegradation performance. As the results, the highest photodegradation performance of Ag-pH10 was achieved due to the high surface area facilitated the adsorption of dyes molecules and the efficient charge (h^+^) separation between heterojunction of semiconductors. On the other hand, the photodegradation performance reduced due to an increase in the amount of Ag_4_P_2_O_7_ (Ag-pH12). The active sites of Ag_3_PO_4_ were more covered by Ag_4_P_2_O_7_. The wide band gap of Ag_4_P_2_O_7_ required the high energy to create electron–hole pairs and the recombination of the electrons in the CB of Ag_3_PO_4_ and the h^+^ in the VB of Ag_4_P_2_O_7_ was increased. Therefore, an appropriate content of Ag_4_P_2_O_7_ is crucial to achieve optimal photocatalytic performance.

**Figure 9:**
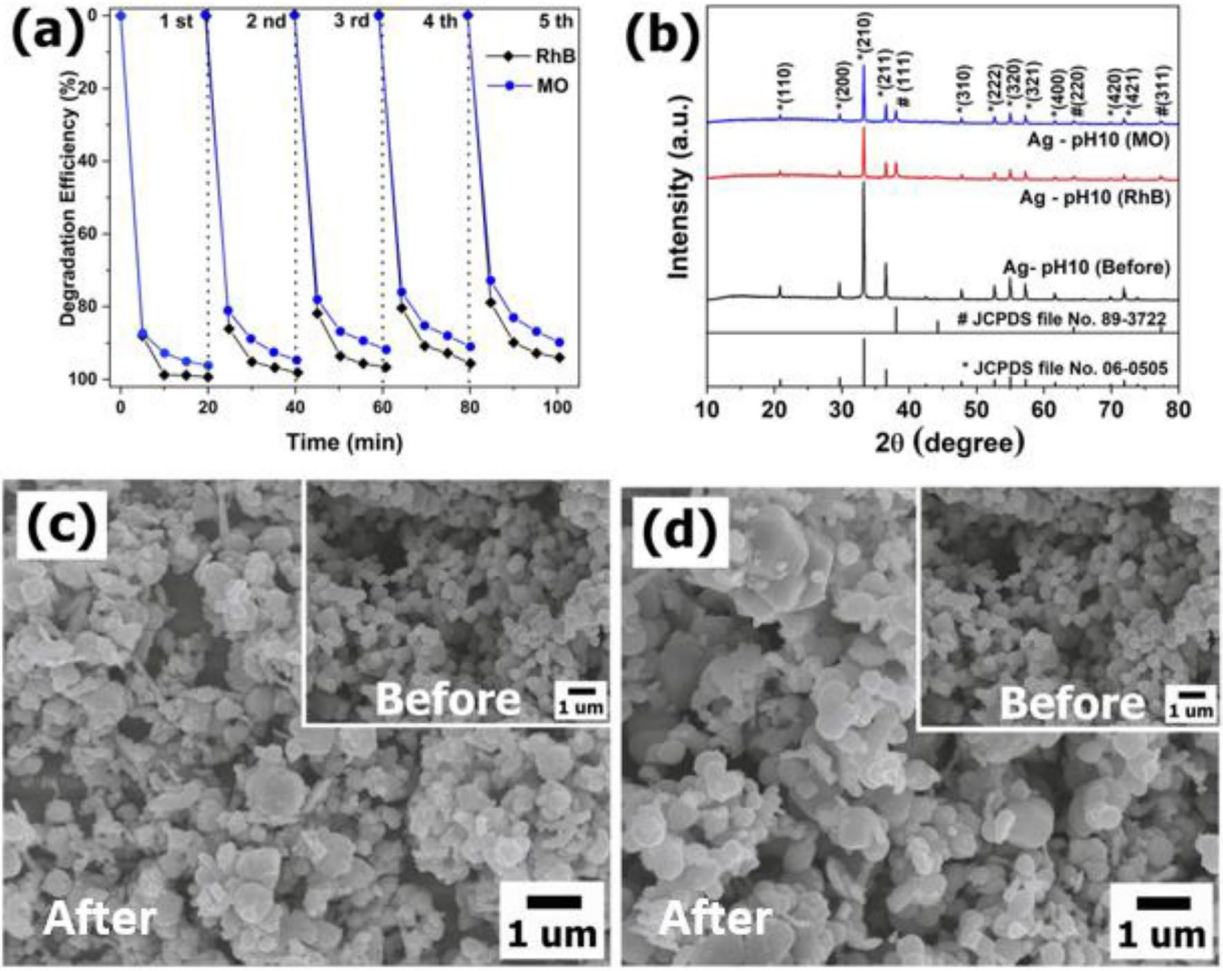
(**a**) Recycled tests for photodegradation and (**b**) XRD pattern of Ag-pH10 before and after photodegradation. SEM image of Ag-pH10 after photodegradation of (**c**) RhB and (**d**) MO (inset shows SEM image of Ag-pH10 before photodegradation as previously shown in Fig. 1e).

Stability of the photocatalyst were extremely significant besides the photocatalytic activity. Figure 9a shows the recycling photocatalytic experiments of Ag-pH10 towards the degradation of RhB for five cycles. It is seen that, the photodegradation performance decreased from one cycle to another. In relation to first cycle run, the degradation efficiency reduced 5.28% for RhB and 6.47% for MO at the end of fifth cycle runs. Thus, the structural and morphological stability of Ag-pH10 after the photocatalysis tests were investigated by XRD and SEM analysis. Figure 9b showed XRD pattens of Ag-pH10 before and after the photocatalysis tests. After fifth cycles, the diffraction peaks of Ag-pH10 were indexed to mixed phase of the cubic Ag_3_PO_4_ phase (JCPDS file No. 06-0505) and cubic Ag phase (JCPDS file No. 89-3722), indicating that crystal structure of Ag_3_PO_4_ was destroyed. Furthermore, the particles size increased after photocatalytic process (Fig. 9c–d). The degradation efficiency decreased because the structural and morphological changed in photocatalyst.

## Conclusion

Ag_3_PO_4_/Ag_4_P_2_O_7_ photocatalysts were successfully synthesized by 270 W microwave-hydrothermal method for 30 min with adjusted pH values by sodium hydroxide and acetic acid. The effect of OH^−^ ions might prevent the agglomeration and producing small particle whereas the acid solution might not suppress the agglomeration and producing a large particle. Thus, the particle size decreased but particle shape was not affected by pH value. Moreover, Ag_4_P_2_O_7_ was found at the surface of particles with high pH value (pH > 7) in precursor solution. As the results, the Ag-pH10 exhibited the highest photodegradation efficiency of RhB of 99.34% and MO of 96.12% within 20 min irradiation due to the highest surface area and the present of optimal Ag_4_P_2_O_7._ Therefore, Ag_3_PO_4_/Ag_4_P_2_O_7_ photocatalyst presented excellent photodegradation performance with high stability and durability. The active species trapping experiments showed that RhB and MO molecules were decomposed by h^+^ and •O_2_^−^ which h^+^ is the main active specie during the photocatalytic process.

## Data Availability

The datasets used and/or analysed during the current study available from the corresponding author on reasonable request.
